# *Mycobacterium chelonae* Wound Infection after Liposuction

**DOI:** 10.3201/eid1607.090156

**Published:** 2010-07

**Authors:** Moon J. Kim, Laurene Mascola

**Affiliations:** Author affiliation: Department of Public Health, Los Angeles, California, USA

**Keywords:** Mycobacterium chelonae, outpatient, infection control, nontuberculous mycobacteria, tuberculosis and other mycobacteria, infection, cosmetic surgery, liposuction, bacteria, letter

**To the Editor:** We recently investigated a case of *Mycobacterium chelonae* abdominal wound infection after liposuction performed under local anesthesia at an outpatient medical office. Our aim was to determine whether other cases of atypical mycobaterial infections had previously occurred after liposuction. *M. chelonae* is widely distributed in soil and water, including tap water. Atypical mycobacterial infections have been associated with skin and soft tissue infections, including infections after cosmetic surgeries, and outbreaks have been documented (*1**–**4*). Previously reported potential sources of liposuction equipment contamination have been inadequate disinfection or sterilization after rinsing of liposuction equipment with tap water, tap water used in cleaning liposuction cannulae, or the quaternary ammonium solution used to disinfect liposuction equipment (*2**,**4*). Increased numbers of procedures performed in freestanding medical centers (not connected with hospitals) that are not routinely monitored by infection control committees or equivalent oversight bodies may contribute to atypical mycobacterial infection (*1*).

Our investigation showed that proper cleaning, disinfection, and sterilization of liposuction equipment and other infection control issues at this medical office were concerns. Except for the physician, only unlicensed medical assistants worked at this office. This staff had been trained to clean and sterilize liposuction equipment, but no written procedures existed for processing reusable liposuction equipment, no logs were kept of autoclave use for sterilization, and preventive maintenance checks and verification of sterility on the autoclave by using biological indicators as recommended by the manufacturer were not performed. The office did not have any general written infection control policies. Office staff mixed leftover solutions from open small bottles of povidone iodine and placed this mixture into larger containers. Staff stored wet alcohol-soaked cotton balls in multiuse containers for wiping tops of multidose vials instead of using individual alcohol prep pads; and 70% isopropyl alcohol solution from an open nonsterile bottle was used instead of sterile irrigation solutions to flush the liposuction suction cannula to dislodge tissue from the ports during the procedure.

Case finding and surveillance of acid-fast bacilli results routinely reported to our public health tuberculosis program did not indicate any other cases of postliposuction wound infections caused by atypical mycobacteria associated with this office. Laboratory testing of environmental samples, including tap water and faucet aerator samples, also did not indicate a source for *M. chelonae* in this outpatient office. This case was likely an isolated occurrence in which the case-patient acquired infection through an environmental source unrelated to this office. However, because of the infection control concerns observed in this office and because the incubation period for *M. chelonae* can be as long as 5 months (*2*), the physician was advised to develop infection control policies and procedures; develop protocols for cleaning, disinfecting, and sterilizing liposuction equipment in accordance with the manufacturer’s recommendations; ensure autoclave sterility by using biological indicators; educate office staff about basic infection control practices and use of aseptic techniques; and notify public health officials of any further infections postliposuction.

Risk factors that cause or contribute to infectious disease outbreaks in outpatient settings include inadequate cleaning, disinfection, sterilization, and storage of instruments and equipment; inappropriate use of barrier equipment, such as gloves, by healthcare personnel; inadequate handwashing practices by healthcare workers; failure to use aseptic techniques; and lack of familiarity with established infection control practices by ambulatory care personnel (*5*). Also, in the outpatient setting, responsibility for implementing an infection control program usually is not assigned to a specific person (*5*), and outpatient medical offices are not routinely monitored by oversight bodies or infection control committees as are hospitals and outpatient surgical centers (*6*).

The California Business and Professions Code requires that outpatient surgery settings using anesthesia, other than local anesthesia or peripheral nerve blocks, be accredited by an oversight body. However, because this facility used only local anesthesia, it did not fall under this code of regulations for facility accreditation, oversight, certification standards, and quality assurance for general public health safety and welfare.

Lack of adherence to basic infection control principles, specifically in outpatient settings, has resulted in outbreaks (*1**,**7**–**10*). Our findings at this medical office further highlight the unaddressed infection control problems in outpatient settings. Because of insufficient oversight for the outpatient setting, professional organizations, state medical boards, and federal and state authorities should consider the need to systematically address infection control standards and monitoring tailored for this setting. As more healthcare procedures move to the outpatient setting, ensuring appropriate infection control practices can prevent outbreaks.

## References

[1] De Groote MA, Huitt G. Infections due to rapidly growing mycobacteria. Clin Infect Dis. 2006;42:1756–63. 10.1086/504381 16705584

[2] Meyers H, Brown-Elliott BA, Moore D, Curry J, Truong C, Zhang Y, An outbreak of *Mycobacterium chelonae* infection following liposuction. Clin Infect Dis. 2002;34:1500–7. 10.1086/340399 12015697

[3] Murillo J, Torres J, Bofill L, Ríos-Fabra A, Irausquin E, Istúriz R, Skin and wound infection by rapidly growing mycobacteria—an unexpected complication of liposuction and liposculpture. Arch Dermatol. 2000;136:1347–52. 10.1001/archderm.136.11.1347 11074697

[4] Centers for Disease Control and Prevention. Rapidly growing mycobacterial infection following liposuction and liposculpture—Caracas, Venezuela, 1996–1998. MMWR Morb Mortal Wkly Rep. 1998;47:1065–7. 9879630

[5] Arias KM. Outbreaks reported in the ambulatory care setting. In: Arias KM, editor. Quick reference to outbreak investigation and control in health care facilities, 1st ed. Sudbury (MA): Jones & Bartlett Publishers; 2000. p. 113.

[6] Lapetina EM, Armstrong EM. Preventing errors in the outpatient setting: a tale of three states. Health Aff. 2002;21:26–39. 10.1377/hlthaff.21.4.26 12117139

[7] Thompson ND, Perz JF, Moorman AC, Homberg SD. Nonhospital healthcare-associated hepatitis B and C virus transmission: United States, 1998–2008. Ann Intern Med. 2009;150:33–9. 19124818 10.7326/0003-4819-150-1-200901060-00007

[8] Watson JT, Jones RC, Siston AM, Fernandez JR, Martin K, Beck E, Outbreak of catheter-associated *Klebsiella oxytoca* and *Enterobacter cloacae* bloodstream infection in an oncology chemotherapy center. Arch Intern Med. 2005;165:2639–43. 10.1001/archinte.165.22.2639 16344422

[9] Rutala WA, Weber DJ. Disinfection and sterilization in health care facilities: what clinicians need to know. Clin Infect Dis. 2004;39:702–9. 10.1086/423182 15356786

[10] Kim MJ, Bancroft E, Lehnkering E, Donlan RM, Mascola L. *Alcaligenes xylosoxidans* bloodstream infections in an outpatient office. Emerg Infect Dis. 2008;14:1046–52. 10.3201/eid1407.070894

